# Associations of Dairy Intake with Arterial Stiffness in Brazilian Adults: The Brazilian Longitudinal Study of Adult Health (ELSA-Brasil)

**DOI:** 10.3390/nu10060701

**Published:** 2018-05-31

**Authors:** Amanda Gomes Ribeiro, José Geraldo Mill, Nágela Valadão Cade, Gustavo Velasquez-Melendez, Sheila Maria Alvim Matos, Maria del Carmen Bisi Molina

**Affiliations:** 1 Centro de Ciências da Saúde, Universidade Federal do Espírito Santo, Vitória CEP 29042-755, Brazil; amandagribeiro@gmail.com (A.G.R.); josegmill@gmail.com (J.G.M.); nagelavc@terra.com.br (N.V.C.); 2 Escola de Enfermagem, Universidade Federal de Minas Gerais, Belo Horizonte CEP 30130-100, Brazil; jguveme@ufmg.br; 3 Instituto de Saúde Coletiva, Universidade Federal da Bahia, Salvador CEP 40110-040, Brazil; sheilaalvim@gmail.com

**Keywords:** dairy, cardiovascular health, pulse wave velocity, arterial stiffness

## Abstract

Recent studies have suggested the possible effect of dairy product intake on cardiovascular risk markers, including arterial stiffness. Our aim was to investigate whether dairy food intake is associated with arterial stiffness, which we assessed by carotid-femoral pulse wave velocity (cfPWV) and pulse pressure (PP) in a cross-sectional analysis of baseline data (2008–2010; *n* = 12,892) of the Brazilian Longitudinal Study of Adult Health (ELSA-Brasil). Dairy consumption was evaluated with a validated food-frequency questionnaire (FFQ) by computing servings per day for total and subgroups of dairy products. Dairy consumption was described in four categories (≤1 serving/day to >4 servings/day). Covariance analysis (ANCOVA) was used to compare cfPWV across increasing intake of dairy food, adjusting for confounding factors, including non-dairy food groups. The intake of total dairy was inversely associated with cfPWV and PP (−0.13 m/s and −1.3 mmHg, from the lowest and to the highest category of dairy intake). Low-fat dairy, fermented dairy and cheese showed an inverse relationship with cfPWV and PP. These findings suggest a beneficial effect of dairy consumption to reduce arterial stiffness. However, further evidence from longitudinal studies or long-term intervention is needed to support reduction of cfPWV and PP mediating the beneficial effects of dairy products on cardiovascular health.

## 1. Introduction

Cardiovascular diseases (CVD) are the leading cause of death worldwide. There were 17.7 million deaths due to CVD in 2015, representing 31% of the total mortality rate recorded in that year. More than 75% of these deaths were recorded in low- and middle-income countries where CVDs most often affect individuals of working age and have a high economic and social impact [1]. According to estimates, 80% of the CVD burden is associated with modifiable behaviors, mainly eating behaviors, that affect risk factors such as arterial hypertension, diabetes mellitus (DM) and obesity [2].

Longitudinal studies suggested an inverse association between dairy product intake and risk of CVD, coronary disease and infarction [3,4]. These findings were consistent with observational studies that showed an inverse relation between milk intake and CVD risk factors such as hypertension [5,6]. The Dietary Approaches to Stop Hypertension (DASH) study was one of the first intervention studies supporting such an inverse relation and showed that diets rich in fruits, vegetables and low-fat dairy products (3 servings/day) were associated with lower systolic (SBP) and diastolic blood pressure (DBP) [7]. A meta-analysis of randomized clinical trials corroborated these findings and showed significant reductions in SBP (by 6.74 mmHg) and DBP (by 3.54 mmHg), values associated with the DASH diet [8].

Recent studies have also suggested the possible effect of dairy product intake on other cardiovascular risk markers, mainly arterial stiffness [9,10]. Stiffening of large arteries is characteristic of the aging process and may influence the development of chronic diseases such as isolated systolic hypertension. Conversely, arterial stiffness can also increase as a consequence of several factors, including type-II diabetes mellitus (DM), obesity and lifestyle characteristics such as smoking, physical activity and diet [11].

Carotid–femoral pulse wave velocity (cfPWV) is accepted as the ‘gold standard’ measurement of arterial stiffness, and it has been used to predict cardiovascular events [12]. There are a limited number of studies investigating the effect of diet on arterial stiffness. Epidemiological studies suggest that dairy may be inversely associated with cfPWV [9,13,14]. A cross-sectional and multi-center study showed a lower cfPWV of 0.10 m/s for every 100-g/day increase in low-fat dairy intake (*p* = 0.011) [13]. In the Maine-Syracuse Longitudinal Study, the frequency of the overall dairy consumption was also inversely associated with cfPWV [9]. The greater consumption of reduced-fat dairy was also correlated with lower cfPWV in a population with type 1 and type 2 diabetes [14].

There have been limited randomized controlled trials examining the effect of dairy on arterial stiffness. A systematic review concerning the effect of dietary interventions on arterial stiffness evidenced the limited, although consistent, beneficial effects of fermented dairy products [15]. More recently, a randomized, cross-over study found that the addition of non-fat dairy products reduced cfPWV and improved endothelial function [16]. On the other hand, in a cohort with diabetes, improving dietary quality by increasing consumption of fruits, vegetables and dairy did not reduce cfPWV, compared with a control group [17].

The aim of the current study was to assess the association between dairy product intake and arterial stiffness in the Brazilian Longitudinal Study of Adult Health (ELSA-Brasil) cohort. The assessment was done by taking into consideration the impact of CVD on the morbidity and mortality profile of middle-income countries such as Brazil; the relatively scarce data about diet and these diseases in such contexts; and evidence of the relation among dairy product intake, cardiovascular events and their risk factors.

## 2. Materials and Methods 

The Brazilian Longitudinal Study of Adult Health (ELSA-Brasil), registered in clinicaltrials.gov as NCT02320461, is a multi-center cohort study designed to investigate the development of chronic diseases, primarily diabetes and cardiovascular diseases, and their risk factors over long-term follow-ups. ELSA-Brasil enrolled and assessed 15,105 civil servants (35–74 years) of five public universities and one research institution from six different cities (Salvador, Belo Horizonte, Rio de Janeiro, São Paulo, Vitoria, and Porto Alegre). Therefore, the sample came from the northeast (13.4% of participants), southeast (72.9% of participants), and southern (13.7% of participants) regions of Brazil. The study was approved by the National Research Ethics Commission (CONEP—976/2006) and by the research ethics committee of each institution. All participants signed an informed consent form.

The baseline procedures (2008–2010) included an interview for recruitment and signature of the informed consent. Clinic exams, blood and urine collection, and application of the questionnaires were done during a single visit to one of the six investigation centers (IC). All subjects were interviewed using a structured interviewer-administered questionnaire that included information on socio-demographic characteristics, lifestyle habits and detailed medical history; on the same day, the dietary intake of nutrients was collected with a validated semi-quantitative food frequency questionnaire (FFQ) [18].

The sample included volunteers and people who were actively recruited from lists of employees provided by the institutions. The exclusion criteria were as follows: Intention to leave the institution; being pregnant or having been pregnant less than four months before; having severe cognitive or communication difficulty and; if retired, living outside the metropolitan region. Sample size was calculated based on estimations of the incidences of type-2 DM and myocardial infarction in the Brazilian population and was compensated for sex differences and possible losses during follow-up. For a better distribution, recruitment goals were defined by sex (50% each), age (15% aged 35–44, 30% aged 45–54, 40% aged 55–64 and 15% aged 65–74 years) and occupational category (35% of support level, with incomplete elementary school; 35% with high school; and 30% with higher education/teaching level) [19].

For the present analysis, baseline data from ELSA-Brasil were used. We excluded participants with self-reported previous cardiovascular disease (*n* = 1001) and bariatric surgery (*n* = 107). Individuals who reported caloric intake <500 or >6000 kcal/day (*n* = 408), and individuals with an unvalidated cfPWV value (*n* = 372) were also excluded, which left 12,892 subjects with complete data for analysis.

### 2.1. Dietary Assessment and Dairy Measurement

Dietary intake was measured by an FFQ that was specific and validated in this population and included 114 food items relevant to the past 12 months [20]. For each food item ascertained, the FFQ included measures of portions and frequency of consumption, the latter of which with 8 response options: >3 times/day, 2–3 times/day, 1 time/day, 5–6 times/week, 2–4 times/week, 1 time/week, 1–3 times/month, and never/almost never.

Cow milk, cheese curds, yogurt, and cheeses were classified as “Milk and Cheese Group”, and butter is described as a dairy product that should be consumed in moderation according to the Dietary Guidelines for the Brazilian Population [21]. In the ELSA FFQ, questions on dairy products included milk (skimmed milk, low-fat milk and whole milk), yogurt (regular, low-fat), cheese (regular, low-fat) and butter.

Servings of specific dairy foods were converted into daily servings, and total daily servings of dairy foods were calculated by summing all dairy foods. For each dairy food, a standard serving size was specified: 240 g for milk, 120 g for yogurt, 30 g for cheese and 5 g for butter.

We computed servings per day for total dairy intake (with butter) and the following dairy subgroups: Full-fat dairy without butter (whole milk, regular yogurt and cheese), low-fat dairy (skimmed milk, low-fat milk, low-fat yogurt and cheese), fermented dairy (total yogurt and cheese), milk, cheese, yogurt and butter.

### 2.2. Pulse Wave Velocity and Blood Pressure

Carotid-femoral pulse wave velocity (cfPWV), pulse pressure (PP) and systolic blood pressure (SBP) were used as dependent variables in the current study.

The cfPWV was measured using an automatic device (Complior, Artech Medical, France) with the subject in the supine position in accordance with the ELSA-Brasil protocols. The distance from the sternal furcula to the right femoral site where the pulse was recorded was measured with a metric tape, regardless of abdominal curvature. Pulse sensors were positioned in the right carotid and femoral arteries, and pulse waves were recorded and visualized on a computer screen. cfPWV was calculated by dividing the distance from the furcula to the femoral pulse by the difference between the delay between the rising phases of the carotid and femoral pulses, and it was expressed in m/s. A subject’s cfPWV was the arithmetic average of readings obtained in ten consecutive cardiac cycles at a regular heart rate. Exams were recorded in each of the six investigation centers by trained and certificated researchers. Training and certification of each investigator was performed by a senior investigator. Validation of all exams obtained at the six investigations centers were performed in a central reading laboratory of cardiovascular physiology [22].

Systolic blood pressure (SBP) was measured using a validated oscillometric device (Omron HEM 705CPINT) after a 5-minute rest with the subject in a sitting position. Three measurements were taken at 1-minute intervals. The mean of the two latest BP measurements was considered as the casual BP. Pulse pressure was calculated as the arithmetic difference between SBP and DBP.

### 2.3. Covariates

Socio-demographic characteristics were included as covariables (potential confounders) of the study: Sex, age (continuous variable; years), race, per capita income (continuous variable); and lifestyle: Alcohol intake (g ethanol/day), physical activity (metabolic equivalent min/week) and smoking status (never smoked, ex-smoker, current smoker).

As adjustment for height when assessing cfPWV has been recommended [23], height, weight and waist circumference were used rather than BMI. Anthropometric measurements were obtained while participants were standing and dressed in a light uniform standardized for the study. We measured body weight to the nearest 0.1 kg with a calibrated scale (Toledo 2096PP) and height with a wall-mounted stadiometer (Seca-SE-216) to the nearest 0.1 cm. Waist circumference was measured using a non-stretchable tape around the midpoint between the lower border of ribs and the iliac crest.

Traditional cardiovascular risk factors with an effect on arterial stiffness were also included: Fasting glycaemia (mg/dL), total cholesterol (mg/dL) and mean blood pressure (MAP, mmHg), calculated as (PAS + (2 × PAD))/3. In addition, the use of antihypertensive, lipid-lowering and antidiabetic drugs (yes/no) were also included.

The following non-dairy food groups (g/day) were considered as possible confounders in our analyses: Fruit, vegetables, whole grains, fish, processed and unprocessed red and white meat.

### 2.4. Statistical Analyses

Data were analyzed with SPSS (Version 18, Chicago, IL, USA). Preliminary analyses were performed to assess correlations between dairy intake, cfPWV and other demographic, health status, and nutrition and lifestyle factors. Participant characteristics in the study were compared according to the dairy intake group (≤1 serving/day, >1–2 servings/day, >2–4 servings/day and >4 servings/day). For continuous variables, analysis of variance (ANOVA) was used. For categorical variables, Chi-square tests were performed.

Covariance analysis (ANCOVA) was used to compare cfPWV across increasing intake of dairy food consumption ranging from ≤1 serving/day to >4 servings/day. Adjustments for multiple comparisons among dairy food intake groups were made and reported in terms of the Bonferroni adjustment. Linear trend was tested by modelling categorical dairy servings per day (≤1 serving/day, >1–2 servings/day, >2–4 servings/day, >4 servings/day) as a continuous variable in the multivariable regression models.

We adjusted for covariates in 4 models as follows: Model 1: Demographic characteristics (age, sex, race, and income); Model 2: Model 1 + anthropometric variables (weight, height, and waist circumference) + lifestyle factors (smoking status, alcohol intake, and physical activity); Model 3: Model 2 + fasting glucose, total cholesterol, MAP (only for cfPWV) and use of drugs (antihypertensive, antidiabetic and lipid-lowering drugs); and Model 4: Model 3 + caloric intake (kcal/d) and non-dairy food groups (g/day; fruit, vegetables, whole grains, fish, and processed and unprocessed red and white meat).

These variables were selected if significantly associated in a simple correlation matrix with dairy food intake (the predictor) or cfPWV (the primary outcome variable), or if known to be related according to previously published studies.

## 3. Results

The median of total dairy product intake was 2.6 servings/day (interquartile range (IQR): 1.43–4.10), 1.00 serving/day of low-fat dairy (IQR: 0.13–2.20), and 1.07 serving/day of whole dairy (IQR: 0.27–2.24). Cheese had the highest median intake in servings/day (1.00, IQR: 0.37–2.00), followed by milk (0.80, IQR: 0.07–1.25). The analysis in grams per day showed that the most consumed dairy product was milk (mean: 235.6 g/day), mainly whole milk (mean: 113.8 g/day), which was followed by cheese (mean: 43.3 g/day) and yogurt (mean: 37.4 g/day). In Table 1, we present the consumption of dairy subgroups according to categories of total dairy intake.

Table 2 summarizes the sociodemographic, health and diet data. The mean (±SD) age was 51.7 ±8.9 years. Dairy product intake was higher in women, as well as among white people. Participants with higher dairy product intake reported lower alcohol intake and smoking habits as well as a higher physical activity level. Groups presenting higher dairy product intake also showed higher per capita family income and lower SBP, DBP, MAP, PP and glycaemia values.

The cfPWV, PP and SBP values decreased as the dairy product intake increased (from ≤1 serving/day to >4 servings/day). Table 3 shows the confidence interval (95%) associated with the mean of the outcomes of each intake group and summarizes the results of the statistical analyses applied to the adjusted models according to the sociodemographic, anthropometric, lifestyle, clinical and dietary variables. The lowest cfPWV values (mean = 9.17 m/s) in the model adjusted for demographic variables were recorded in the group presenting the highest dairy product intake (>4 servings/day). Such a trend remained in the models adjusted for anthropometric, lifestyle, clinical and dietary variables.

**Figure 1:**
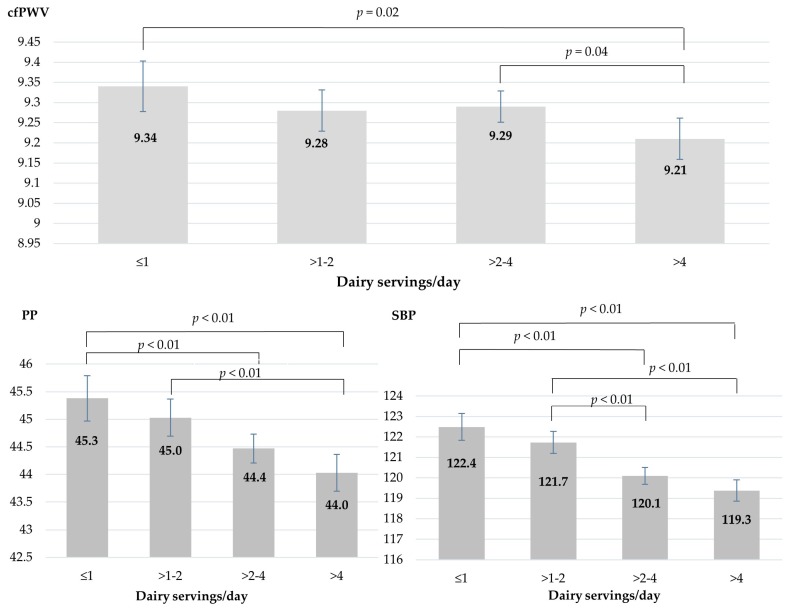
Mean values of carotid-femoral pulse wave velocity (cfPWV), pulse pressure (PP) and systolic blood pressure (SBP) in dairy intake groups after adjustment for variables in Model 4.

The cfPWV values in the lowest intake category (≤1 serving/day) were significantly higher than those in the highest intake category (*p* = 0.02). Comparisons between intake categories showed that participants who consumed more than 4 servings of dairy products/day presented lower PP and SBP values than those who consumed less than 1 serving/day or between 1 and 2 servings/day after the model was adjusted to avoid possible confounding factors (Figure 1).

Table 4 shows differences in cfPWV, SBP and PP values according to the intake (servings/day) of different dairy subgroups. Low-fat dairy products, fermented dairy, and cheese showed a significant inverse association with cfPWV and PP. Whole and low-fat dairy products, fermented dairy, milk, and cheese showed a significant inverse association with SBP. Butter showed a significant inverse association only with cfPWV.

## 4. Discussion

The intake of dairy products in the current study was inversely associated with cfPWV, PP and SBP, adjusted by sociodemographic, anthropometric, lifestyle, clinical and other dietary factors. The cfPWV value was significantly lower in participants presenting a higher intake of dairy products than in those classified in the lowest intake category. These results were supported by SBP and PP findings (replacing haemodynamic indices used to assess arterial stiffness), which linearly decreased as the dairy product intake increased. Recent epidemiological findings suggested an inverse association between dairy product intake, particularly fermented dairy, and CVD [4]. One of the possible mechanisms of the beneficial effect of dairy products on cardiovascular health lies with the inverse relation between the consumption of these products and BP [24] and, possibly, between these products and arterial stiffness, which can be evaluated by measuring the PWV (as in the present study) or by the augmentation index (AI)) obtained in arterial tonometry [9,10,13].

Dairy intake was associated with lower BP and lower risk of developing hypertension in observational studies [24], but randomized controlled trials have shown mixed results [16,25]. Meta-analysis of seven mostly short-term randomized controlled studies with 711 adults found no significant effects of increased dairy food on the BP. However, most of the trials were small and of modest quality [25].

The current results corroborate with observational studies assessing the relation between dairy product intake and measures of arterial stiffness in populations in the United States and Wales. Crichton et al. [9] found lower cfPWV values in individuals consuming dairy products more than 5–6 times per week than in individuals presenting lower regular intake (1–4 times per week). In an analysis based on data from the Caerphilly Prospective Study, Livingstone et al. [10] observed that the AI was 1.8% lower in individuals in the highest dairy product intake quartile, after a 22.8-year follow-up, but no association was found for cfPWV. On other hand, Petersen et al. [14] found an inverse association between higher dairy intake and cfPWV, but not AI, in a cohort with diabetes. Previous research supports a dissociation between cfPWV and AI, and this phenomenon may be modulated by the presence of several factors, e.g., aging, use of medicaments and insulin resistance [26].

Intervention trials examining the effect of dairy on arterial stiffness are lacking. A randomized, cross-over study showed that an additional 4 servings of non-fat dairy per day reduced cfPWV compared with the no-dairy condition (4 servings of fruit juice). However, fruit juice may have a detrimental effect on arterial stiffness and the result could be a reflection of this [16]. In a cohort of individuals with type 1 and type 2 diabetes, AI and cfPWV were not improved with fruit and dairy increased in the intervention group after 12 months. However, the result may have been limited by the poor compliance of the participants with the intervention [17].

The mechanisms by which dairy products could reduce BP and arterial stiffness are not yet fully understood. However, it is hypothesized that bioactive peptides released during milk-protein digestion and milk-fermentation processes may be involved in this relation. Peptides deriving from casein were capable of reducing the action of the angiotensin converting enzyme (ACE) in experimental models, and it could reduce the circulating angiotensin II levels, thus preventing vasoconstriction and oxidative stress and increasing endothelium-dependent vasorelaxation [27,28]. Furthermore, there is evidence that certain milk peptides can inhibit the release of the vasoconstrictor endothelin-1 by endothelial cells, thus avoiding increased BP [29]. Randomized clinical trials reported the benefit of fermented dairy products rich in casein [30] and whey [31] on reducing BP and arterial stiffness.

A variety of other biologically active components, such as calcium, potassium and magnesium in dairy products, may also have an impact on BP and arterial stiffness [28]. Several possible mechanisms associated with the role played by calcium in cardiovascular health have been investigated. There is evidence that calcium can lower BP by regulating the renin-angiotensin system and by improving the sodium-potassium balance in humans [32]. A consistent set of results recorded in observational studies, clinical trials and meta-analyses indicated that high dietary potassium intake is associated with lower BP. Dietary potassium intake is important because it leads to vasodilation through sodium-potassium pump stimulation and through the opening of potassium channels. In addition to vasodilation, many other mechanisms by which potassium can influence BP, such as natriuresis, changes in intracellular sodium and tonicity, baroreceptor sensitivity modulation, and reduced sensitivity to norepinephrine and angiotensin II, have been investigated [33]. For magnesium, several mechanisms such as calcium channel blockade, competition with sodium for binding sites in vascular smooth muscle cells, increased prostaglandin E, endothelium-dependent vasodilatation and endothelial dysfunction improvement may also be involved in BP regulation. Magnesium is the most effective in reducing BP when it is used in combination with calcium and potassium [34].

In the present study, fermented dairy was inversely associated with cfPWV, PP and SBP values. However, the individual analysis applied to the dairy products showed that only for cheese this inverse association was found. Such a finding supports the result of a recent dose-response meta-analysis that assessed the relation between fermented dairy product intake and the risk of developing CVD. Cheese (10 g/day) was marginally inversely related to CVD (RR: 0.98; 95% CI: 0.95–1.00; 11 populations), whereas no significant association was found between yogurt and CVD [35].

Furthermore, we found an inverse association between the intake of low-fat dairy products and cfPWV, corroborating with observational [13] and interventional [16] studies. Recio-Rodriguez et al. found a decrease in cfPWV of 0.10 m/s estimated for every 100 g/day increase in low-fat dairy intake. However, the authors also found a cfPWV increase by 0.11 m/s estimated for each 100-g/day increase in the intake of whole dairy products. In the present study, there was no statistically significant association between the intake of whole dairy products (without butter) and cfPWV, and there was a smaller but significant association with SBP, comparing with low-fat dairy products. One hypothesis for the different results presented between full-fat and low-fat dairy in BP could be the bioavailability of minerals. It is possible that when consumed with fatty acids, released from the diet during digestion in the small intestine, the divalent cations of calcium and magnesium form insoluble soaps not absorbed by enterocytes [36].

In sensitivity analyses, we investigated whether intake of cheese was driving the associations with cfPWV for low-fat dairy intake by excluding this item from this variable. As a result, the association was not found for the subgroup with skimmed milk, low-fat milk and, low-fat yogurt, but the significant association remained for low-fat cheese alone (*B* = −0.04, *p* < 0.001). The same result was not found for full-fat cheese.

Interestingly, butter was inversely associated with cfPWV, a finding not detected for the other haemodynamic indexes. Previous analysis in the ELSA-Brasil population showed a graded inverse association between full-fat dairy and butter consumption and metabolic syndrome, and the results suggested that saturated fatty acids (SFAs) found in dairy products could be responsible for this association [37]. Saturated fat consumption has been classically related to elevated LDL (plasma cholesterol) and to increased cardiovascular risk. However, prospective cohort studies and clinical trials investigating the association between milk fat and CVD did not find clear evidence on the intake of whole dairy products and CVD [38,39]. According to a recent meta-analysis, the intake of high-fat dairy products (around 200 g/day) did not show an association with CVD (RR: 0.93; 95% CI: 0.84–1.03) [35]. A prospective study investigating the association between the intake of different dietary sources and the incidence of cardiovascular events in the Multi-Ethnic Study of Atherosclerosis (MESA) population found that a higher intake of saturated fat from dairy products was associated with a lower incidence of CVD. Replacement of 2% of the energy of the saturated fat derived from meat by the energy of the saturated fat derived from milk was associated with 25% less risk of developing CVD [40]. Moreover, in two prospective cohorts, higher plasma dairy fatty acid concentrations were associated with lower incident diabetes [41]. However, in the present study inverse association was only found between cfPWV and butter, but not for whole fat dairy (milk, cheese, and yogurt). It is possible that butter consumption has been acting as a marker for an unknown confounding variable.

The differences in cfPWV and SBP associated with increased dairy product intake were relatively small in the current study, 0.13 m/s for cfPWV and 3.1 mmHg for SBP between the lowest and the highest intake category. Even though they are small, these differences may be clinically relevant. Meta-analysis of the predictive cfPWV value in cardiovascular and death events estimated a cfPWV increase of 1 m/s represents a 14%, 15% and 15% increase in the risk (adjusted for age and sex) of cardiovascular events, death by CVD, and all-cause mortality, respectively [42]. According to Selmer et al. [43], the reduction of 4 mmHg in SBP would be equivalent to a 15.7% lower risk of death by stroke and to a 9.9% lower risk of myocardial infarction. Therefore, our study supports the view that reduction of cfPWV and BP can mediate the beneficial effects of dairy food on cardiovascular outcomes.

Our study has strengths and limitations. It is the first study to examine the relationship between dairy food intake and arterial stiffness, as measured by cfPWV, in Latin America. Moreover, this relationship was examined in a large sample (12,892 individuals from both sexes) with a wide age range and controlled for relevant demographic, health and dietary variables. However, the cross-sectional and observational nature of the study has limitations to be considered, since the findings may result from residual confusion despite the extensive adjustment for other food variables and confounding factors. The cross-sectional nature of the study, with a single measure of cfPWV and BP, does not allow conclusions regarding causality.

In addition, although FFQs have been widely adopted in epidemiological studies, the method also presents limitations, mainly because it produces a retrospective and subjective diet evaluation. FFQ depends on participant memory, and it may lead to reports of insufficient or excessive food intake. Another limitation was the absence of any objective biomarker of dairy intake, e.g., levels of pentadecanoic and margaric acid levels.

## 5. Conclusions

Higher dairy food intake was associated with lower cfPWV and accompanying lower PP and SBP values. However, dairy food intake was associated with many confounders that are generally associated with better health, such as less smoking status, higher physical activity and fruit and vegetable intake, and higher socioeconomic status. Therefore, additional evidence from longitudinal and randomized studies are necessary to conclude if reduction of arterial stiffness and BP effectively mediate the beneficial effects of dairy products on cardiovascular health, thus preventing development of CVD.

## Figures and Tables

**Table 1:** Intake of dairy products according to categories of total dairy intake. ELSA-Brasil, 2008–2010 ^1^.

Dairy Products, Servings/Day	Categories of Dairy Consumption (Servings/Day)				
	≤1 (*n* = 2036)	>1–2 (*n* = 2862)	>2–4 (*n* = 4700)	>4 (*n* = 3294)	*p*
**Dairy ^2^**	**0.52**	**1.53**	**2.90**	**5.49**	
**Low-fat dairy ^2^**	**0.10**	**0.60**	**1.27**	**2.80**	
Skimmed milk, low-fat milk	0.06 ± 0.18	0.24 ± 0.40	0.52 ± 0.79	1.00 ± 1.34	<0.001
Low-fat yogurt	0.02 ± 0.09	0.08 ± 0.23	0.15 ± 0.35	0.21 ± 0.45	<0.001
Low-fat cheese	0.13 ± 0.19	0.36 ± 0.39	0.77 ± 0.75	1.79 ± 1.77	<0.001
**Full-fat dairy ^2^**	**0.23**	**0.93**	**1.40**	**2.80**	
Whole milk	0.12 ± 0.23	0.30 ± 0.43	0.49 ± 0.79	0.82 ± 1.37	<0.001
Regular yogurt	0.04 ± 0.11	0.11 ± 0.23	0.20 ± 0.38	0.30 ± 0.54	<0.001
Regular cheese	0.14 ± 0.19	0.32 ± 0.37	0.56 ± 0.64	1.22 ± 1.42	<0.001
**Fermented dairy ^2^**	**0.23**	**0.90**	**1.67**	**3.20**	
Yogurt (regular, low-fat)	0.06 ± 0.13	0.19 ± 0.29	0.35 ± 0.45	0.52 ± 0.61	<0.001
Cheese (regular, low-fat)	0.26 ± 0.26	0.68 ± 0.47	1.32 ± 0.84	3.00 ± 1.99	<0.001
Butter	0.04 ± 0.13	0.12 ± 0.28	0.26 ± 0.52	0.67 ± 1.08	<0.001

^1^ ELSA-Brasil, Brazilian Longitudinal Study of Adult Health. Unless otherwise specified, all values are means ±SDs. ^2^ Values are medians.

**Table 2:** Baseline characteristics by categories of dairy consumption of Brazilian participants: ELSA-Brasil, 2008–2010 ^1^.

Characteristics of Participants	Categories of Dairy Consumption (Servings/Day)				*p*
	≤1 (*n* = 2036)	>1–2 (*n* = 2862)	>2–4 (*n* = 4700)	>4 (*n* = 3294)	
Age, years	51.2 ± 8.3	51.3 ± 8.9	51.7 ± 9.0	52.3 ± 9.2	<0.001
Sex, *n* (%)					
Men	1111 (54.6)	1356 (47.4)	1924 (40.9)	1339 (40.9)	
Women	925 (45.4)	1506 (52.6)	2776 (59.1)	2001 (59.1)	<0.001
Race, *n* (%)					
White	845 (41.5)	1368 (47.8)	2646 (56.3)	1963 (59.6)	
Other	1191 (58.5)	1494 (52.2)	2054 (43.7)	1331 (40.4)	<0.001
Educational level, *n* (%)					
Completed secondary school	872 (42.8)	1081 (37.8)	1516 (32.3)	923 (28.0)	
University degree	728 (35.8)	1399 (48.9)	2743 (58.4)	2133 (64.8)	<0.001
Weight, kg	73.1 ± 14.2	73.7 ± 14.6	72.6 ± 14.7	73.2 ± 15.1	0.019
BMI, kg/m^2^	26.8 ± 4.5	27.0 ± 4.6	26.7 ± 4.6	26.8 ± 4.6	0.111
Waist circumference, cm	91.1 ± 12.2	91.1 ± 12.3	90.1 ± 12.5	90.4 ± 12.7	0.001
Smoking status, *n* (%)					
Never smoker	1033 (50.7)	1642 (57.4)	2829 (60.2)	1980 (60.1)	
Ex-smoker	631 (31.0)	830 (29.0)	1361 (29.0)	943 (28.6)	
Current smoker	372 (18.3)	390 (13.6)	510 (10.8)	371 (11.3)	<0.001
Alcohol intake, g ethanol/day	71.8 ± 142.7	55.7 ± 118.3	47.0 ± 91.2	47.2 ± 90.0	<0.001
Physical activity, min/week	467.1 ± 884.2	557.5 ± 954.0	623.8 ± 1051.8	725.2 ± 1158.7	<0.001
cfPWV, m/s	9.51 ± 1.90	9.33 ± 1.81	9.22 ± 1.79	9.17 ± 1.74	<0.001
Systolic blood pressure, mm Hg	123.7 ± 18.7	122.0 ± 17.5	119.4 ± 16.5	119.2 ± 15.8	<0.001
Diastolic blood pressure, mm Hg	78.0 ± 11.3	77.0 ± 10.9	75.2 ± 10.3	75.1 ± 10.2	<0.001
Mean blood pressure, mm Hg	95.4 ± 12.9	94.0 ± 12.0	91.9 ± 11.4	91.8 ± 11.3	<0.001
Pulse pressure, mm Hg	45.8 ± 11.5	45.0 ± 10.7	44.2 ± 10.3	44.1 ± 9.9	<0.001
Fasting glucose, mg/dL	114.5 ± 35.6	111.3 ± 29.1	110.0 ± 27.8	109.6 ± 26.8	<0.001
Total cholesterol, mg/dL	218.0 ± 43.4	215.9 ± 41.7	214.8 ± 41.3	214.7 ± 41.0	0.019
Drugs, *n* (%)					
Antidiabetic drugs	170 (8.3)	217 (7.6)	334 (7.1)	231 (7.0)	0.247
Lipid-lowering drugs	208 (10.2)	318 (11.1)	572 (12.2)	403 (12.2)	0.066
Antihypertensive drugs	561 (27.6)	813 (28.4)	1235 (26.3)	842 (25.6)	0.055
Food groups, g/day					
Fruit	460.7 ± 405.3	504.3 ± 407.9	548.6 ± 401.8	616.9 ± 452.2	<0.001
Vegetables	191.1 ± 148.8	198.5 ± 136.7	217.5 ± 142.1	240.9 ± 159.4	<0.001
Unprocessed meat	153.7 ± 126.7	161.0 ± 112.4	165.6 ± 114.3	179.3 ± 124.5	<0.001
Processed meat	18.7 ± 23.2	20.1 ± 22.6	21.3 ± 23.0	27.1 ± 28.6	<0.001
Fish	46.8 ± 61.2	50.5 ± 61.3	50.2 ± 58.5	52.2 ± 59.9	0.016
Whole grains	30.4 ± 68.7	37.3 ± 71.1	44.6 ± 70.3	52.6 ± 77.0	<0.001

^1^ Values are presented as the mean ±SD unless otherwise indicated.

**Table 3:** Adjusted cfPWV, PP and SBP means ^1^ according to dairy servings per day among Brazilian adults: ELSA-Brasil, 2008–2010.

Outcome		Categories of Dairy Consumption (Servings/Day)								*p* ^2^
		≤1		>1–2		>2–4		>4		
		*n* = 2036		*n* = 2862		*n* = 4700		*n* = 3294		
		Mean	95% CI	Mean	95% CI	Mean	95% CI	Mean	95% CI	
**cfPWV**	Model 1	9.43	9.36–9.49	9.33	9.27–9.38	9.26	9.22–9.31	9.17	9.12–9.22	<0.001 ^‡^
	Model 2	9.43	9.36–9.49	9.32	9.26–9.38	9.27	9.22–9.31	9.17	9.12–9.22	<0.001 ^‡^
	Model 3	9.34	9.28–9.40	9.28	9.23–9.33	9.29	9.25–9.34	9.21	9.16–9.26	0.006 ^‡^
	Model 4	9.34	9.27–9.40	9.28	9.23–9.33	9.29	9.26–9.34	9.21	9.16–9.26	0.014 ^‡^
**PP**	Model 1	45.3	44.9–45.7	44.9	44.6–45.3	44.4	44.2–44.7	44.1	43.8–44.5	<0.001 ^‡^
	Model 2	45.3	44.8–45.7	44.9	44.6–45.3	44.4	44.2–44.7	44.2	43.9–44.5	<0.001 ^‡^
	Model 3	45.1	44.7–45.5	44.9	44.5–45.2	44.4	44.2–44.7	44.3	44.0–44.6	0.003 ^‡^
	Model 4	45.3	44.9–45.8	45.0	44.7–45.3	44.4	44.2–44.7	44.0	43.7–44.4	<0.001 ^‡^
**SBP**	Model 1	122.4	121.7–123.0	121.6	121.1–122.2	119.9	119.5–120.4	119.6	119.1–120.1	<0.001 ^‡^
	Model 2	122.2	121.6–122.9	121.5	121.0–122.1	120.0	119.6–120.4	119.7	119.2–120.2	<0.001 ^‡^
	Model 3	122.0	121.3–122.6	121.4	120.9–121.9	120.0	119.6–120.5	119.9	119.4–120.5	<0.001 ^‡^
	Model 4	122.4	121.8–123.1	121.7	121.2–122.2	120.1	119.7–120.5	119.3	118.8–119.9	<0.001 ^‡^

^1^ cfPWV, carotid-femoral pulse wave velocity; MAP, mean arterial pressure; PP, pulse pressure; and SBP, systolic blood pressure. Adjusted mean determined by ANCOVA for each of the following variables: Model 1: demographic characteristics (including age (continuous variable; years), sex, race, income (continuous variable. R$)); Model 2: model 1 + anthropometric measurements (weight (kg), height (m), waist circumference (cm)), lifestyle habits (smoking status, alcohol intake (grams of ethanol per day), physical activity (metabolic equivalent min/week)); Model 3: model 2 + fasting glucose (mg/dL), total cholesterol (mg/dL), MAP (mmHg), antidiabetic drugs (yes/no), lipid-lowering drugs (yes/no), antihypertensive drugs (yes/no); Model 4: extended set 2 + dietary (calorie intake (kcal/day) and non-dairy food groups (g/day)). ^2^ *p* for F-test. ^‡^ *p* <0.01 for statistically significant linear trend. Linear trend was tested by modelling dairy servings per day (≤1 serving/day, >1–2 servings/day, >2–4 servings/day and >4 servings/day) as a continuous variable in the multivariable regression models.

**Table 4:** Adjusted differences in cfPWV, PP and SBP associated with a 1 serving/day increase in the intake of dairy products: ELSA-Brasil, 2008–2010 ^1^.

Subgroups of Dairy, Servings/Day ^1^	cfPWV (m/s)	PP (mmHg)	SBP (mmHg)
Low-fat dairy	−0.02 (−0.04, −0.01)	−0.3 (−0.35, −0.15)	−0.4 (−0.58, −0.26)
Full-fat dairy (without butter)	−0.00 (−0.02, 0.01)	−0.0 (−0.16, 0.06)	−0.2 (−0.40, −0.06)
Fermented dairy	−0.02 (−0.04, −0.01)	−0.3 (−0.43, −0.21)	−0.5 (−0.66, −0.33)
Milk	0.01 (−0.01, 0.03)	−0.0 (−0.19, 0.12)	−0.4 (−0.61, −0.12)
Cheese	−0.02 (−0.04, −0.01)	−0.4 (−0.47, −0.24)	−0.5 (−0.69, −0.33)
Yogurt	−0.02 (−0.07, 0.03)	−0.1 (−0.47, 0.24)	−0.5 (−1.05, 0.08)
Butter	−0.05 (−0.09, −0.02)	0.0 (−0.22, 0.25)	−0.1 (−0.51, 0.24)

^1^ Values are presented as the mean (95% CI) adjusted by using multivariable linear regression for variables in model 4.
